# Development of Highly Sensitive and Specific mRNA Multiplex System (XCYR1) for Forensic Human Body Fluids and Tissues Identification

**DOI:** 10.1371/journal.pone.0100123

**Published:** 2014-07-03

**Authors:** Yan Xu, Jianhui Xie, Yu Cao, Huaigu Zhou, Yuan Ping, Liankang Chen, Lihua Gu, Wei Hu, Gang Bi, Jianye Ge, Xin Chen, Ziqin Zhao

**Affiliations:** 1 Department of Forensic Medicine, Shanghai Medical College, Fudan University, Shanghai, China; 2 Shanghai Key Laboratory of Crime Science Evidence, Key Laboratory of Forensic Evidence and Science Technology, Ministry of Public Security, Institute of Forensic Science, Shanghai Public Security Bureau, Shanghai, China; 3 Human Identification Division, Life Technologies, South San Francisco, California, United States of America; Xi'an Jiaotong University School of Medicine, China

## Abstract

The identification of human body fluids or tissues through mRNA-based profiling is very useful for forensic investigations. Previous studies have shown mRNA biomarkers are effective to identify the origin of biological samples. In this study, we selected 16 tissue specific biomarkers to evaluate their specificities and sensitivities for human body fluids and tissues identification, including porphobilinogen deaminase (PBGD), hemoglobin beta (HBB) and Glycophorin A (GLY) for circulatory blood, protamine 2 (PRM2) and transglutaminase 4 (TGM4) for semen, mucin 4 (MUC4) and human beta defensin 1(HBD1) for vaginal secretion, matrix metalloproteinases 7 and 11 (MMP7 and MMP11) for menstrual blood, keratin 4(KRT4) for oral mucosa, loricrin (LOR) and cystatin 6 (CST6) for skin, histatin 3(HTN3) for saliva, statherin (STATH) for nasal secretion, dermcidin (DCD) for sweat and uromodulin (UMOD) for urine. The above mentioned ten common forensic body fluids or tissues were used in the evaluation. Based on the evaluation, a reverse transcription (RT) PCR multiplex assay, XCYR1, which includes 12 biomarkers (i.e., HBB, GLY, HTN3, PRM2, KRT4, MMP11, MUC4, DCD, UMOD, MMP7, TGM4, and STATH) and 2 housekeeping genes [i.e., glyceraldehyde-3-phosphate dehydrogenase (GAPDH) and 18SrRNA], was developed. This assay was further validated with real casework samples and mock samples (with both single source and mixture) and it was approved that XCYR1 is effective to identify common body fluids or tissues (i.e., circulatory blood, saliva, semen, vaginal secretion, menstrual blood, oral mucosa, nasal secretion, sweat and urine) in forensic casework samples.

## Introduction

Short Tandem Repeat (STR) DNA typing, as a routine practice worldwide, is an effective way to identify individuals from crime scene biological samples. However, the identification of the cellular origin of different crime-related body fluids or tissues cannot be done with STR typing, in spite that it is of particular importance for the crime scene reconstruction. Conventional methods of biological stains identification, which use protein or enzyme and rely merely on a color-forming reaction, may be lack of sensitivity and not highly specific in certain scenarios. Traditional blood tests, such as benzidine test and FOB strips, are based on peroxidase-like activity and antigen-antibody immune response of haemoglobin. Although these tests can show positive reaction for human blood, they cannot further demonstrate whether a sample is vaginal blood or circulatory blood, which in fact is very important implication for sexual assault cases. The presumptive test for semen is to detect prostate-specific antigen (PSA), unfortunately PSA can also be detected in vasectomized men and adult male urine [1], [2]. Thus, conventional methods of body fluids identification are presumptive tests but not confirmatory tests. Besides, no tests are available for identifying vaginal secretion, menstrual blood, sweat and urine. On the other hand, a forensic stain may be a mixture of two or more body fluids, such as blood stain and semen from one or more persons, which is unlikely to be identified by naked eyes.

Although it is well known that mRNA is highly unstable and rapidly degraded by ubiquitous RNases, some reports indicated that mRNA in stains is highly stable not only under controlled conditions but when exposed to a range of environmental conditions [3]–[6]. Studies have been done to investigate mRNA profiling as a novel method to identify body fluids [3], [7]–[13] and probably replace traditional serology [4], [11], [13]–[15]. Further, cell-specific mRNA expression levels were compared to demonstrate body fluid stains identification with mRNA profiling [2], [4]–[7], [9], [16], [17]. In addition, since forensic casework materials are often with limited quantities, the ability of mRNA profiling dealing with the samples with co-existing DNA/RNA shows significant advantage over conventional methods [18]–[21].

The main goals of this study were to evaluate the effectiveness of the mRNA markers for human tissue identification and develop a highly sensitive and specific mRNA multiplex system. To achieve these goals, 14 previously identified markers were initially evaluated [4]–[6], [10], [14], [15], [22], which includes HBB, GLY and PBGD (circulatory blood), PRM2 and TGM4 (semen), HBD1 and MUC4 (vaginal secretion), MMP7 and MMP11 (menstrual blood), HTN3 (saliva), KRT4 (oral mucosa), STATH (nasal secretion), LOR and CST6 (skin). We also tested a human antibiotic peptide, dermcidin (DCD), and the Tamm-Horsfall protein, uromodulin (UMOD), since DCD and UMOD are associated with sweat secretion [23]–[25] and urine [26]–[28]. Ten common forensic body fluids or stains (i.e., circulatory blood, saliva, semen, vaginal secretion, menstrual blood, oral mucosa, nasal secretion, skin, sweat, and urine) were used in evaluating the mRNA profiling for body fluid identifications. Among these tested body fluids, particular attentions and efforts were paid on sweat and urine because little studies have been done for these two fluids and they are very common in crime scenes and very important for forensic investigations. Finally, based on the evaluation study, a novel multiplex assay (XCYR1) was developed to detect circulatory blood, saliva, semen (with and without spermatozoa), menstrual blood, vaginal secretion, nasal secretion, oral mucosa, sweat and urine samples, with high specificity and repeatability after primer optimization.

## Materials and Methods

### 1. Ethical statement

This study was approved by the Ethics Committee of Fudan University, China. All the participants provided their written informed consent for the collection of the samples and the subsequent analysis. Subjects are protected by informed consent process – they are informed of what is being collected and repeatedly given the option to withdraw their consent and discontinue their participation. All children in the study had written consent from parents, caretakers, or guardians to participate in the study. The investigation was conducted in accordance with humane and ethical research principles of Fudan University, China. The experiment involving animals was approved by the Experimental Animal Ethics Committee, Fudan University Shanghai Medical College. Animal blood collection was carried out by Shanghai Entry-Exit Inspection and Quarantine Bureau in accordance with the recommendations of the Care and Use of Laboratory Animals from China Council on Animal Care.

### 2. Sample collection

#### 2.1 Human samples

All body fluids were randomly collected from healthy volunteers. Ten individuals donated circulatory blood (short for “blood” in the text below) samples. Eight individuals provided in conjunction with saliva, oral and nasal swab samples. Semen samples were collected from a frozen aliquot of six individuals (four fertile men and two vasectomized men). Semen-free vaginal secretion samples were donated from five individuals and semen-free menstrual blood (day 2 or 3 of the menstrual cycle) samples were donated from five individuals. Eight male individuals (including two boys–4 and 7 years old) and eight female individuals (including two girls–5 and 9 years old) supplied urine respectively. Six individuals' sweat drops from mainly the faces and arms were collected after exercising or bathing and 50 µL sweat samples were used for mRNA extraction. Skin samples from six healthy individuals were collected using sanitary cotton swabs (Pigeon, Japan) from the forehead, the neck, the palm of the hand, and on the sole of the foot by scrapping or rubbing. 10 µL aliquots of fresh circulatory blood or frozen semen and 20 µL of saliva were pipetted on cotton swabs and dried at room temperature. Vaginal secretion and nasal secretion was collected on sterile cotton swabs from the vagina and nasal, respectively, and dried at room temperature. Tongue scrapings were collected on sterile cotton swabs and referred to oral mucosa in the rest of the text. They were used as the general mucosa samples to assess the expression of the general mucosa markers [4]. Menstrual blood was collected on sanitary towels and cut into 2×2 cm^2^ squares for mRNA extraction. For urine samples, 2 mL of urine from each donor was spotted onto cotton cloth (20 cm in diameter) and let it dry at room temperature. The urine stains were cut into 2.5×2.5 cm^2^ squares (corresponding to 40 µL urine sample) and used for mRNA extraction. All body fluids were dried overnight at room temperature and then processed immediately or stored at −80°C.

#### 2.2 Mixture samples

For analyzing blood-semen, oral mucosa- semen, saliva-sweat, menstrual blood-semen, sweat-semen-female urine and blood-sweat mixture samples, samples were mixed at 2∶1 for blood-semen, oral mucosa-semen, menstrual blood-semen mixtures, in a total of 30 µL, or 1∶9 for saliva-sweat mixture, 1∶49 for blood-sweat mixture in a total of 50 µL, 40∶1∶79 for sweat-semen-female urine in a total of 60 µL. Mixed body fluids were obtained by aliquoting volumes of each fluid (from 4 individuals) in ratios of 99∶1, 9∶1 and 1∶1 in a total volume of 30 µL for sweat and blood. Semen and blood were mixed at 1∶1 and 1∶9 in a total volume of 30 µL.

#### 2.3 Mock case sample preparation

Mock case traces were prepared to mimic crime scene biological evidence. The researchers who prepared the samples were not involved in sample analysis. Eight volunteers provided samples used in mock cases. Donors gave informed consent and presented reference DNA profiles. Mock case 1 involved a dried menstrual blood stain (3×3 cm^2^ squares) on the towel, and then 2 µL of semen sampled from a fertile donor was added. Mock case 2, a nasal secretion sample from a donor was collected using a dry sanitary cotton swab and 2 µL peripheral blood from the same donor was collected after a finger prick. Mock case 3 consisted of 10 µL saliva and 2 µL semen from the same donor on the tissue paper. Mock case 4, cell material from a blood swab (2 µL peripheral blood collected with a dry swab) was transferred to a sweat swab (from the forehead collected with a water-moistened swab) of the same donor by rubbing the swabs together. Mock case 5, 5 µL saliva was dropped on a dry swab and was transferred to a sweat swab (from the neck collected with a water-moistened swab) of the same donor by rubbing the swabs together. Mock case 6, the sweat sample was collected from the forehead using a dry sanitary cotton swab, and 20 µL urine sample from a different donor was dropped on the same cotton swab.

For real case work samples, the storage time of the samples range from 2 days to 2 years.

#### 2.4 Animal blood samples

Blood samples from chicken, duck, dog, cat, mouse, pig, cattle, goat and fish were obtained from Shanghai Entry-Exit Inspection and Quarantine Bureau. 100 µL blood was obtained for each species. All blood samples were placed on sterile fine cotton swab and left to dry overnight.

### 3. RNA/DNA co-isolation

RNA was extracted from blood, saliva, semen, mucosa, vaginal secretion and menstrual blood using a column-based extraction kit (Qiagen AllPrep DNA/RNA Mini kit), according to the manufacturer's protocol.

Casework samples and other body fluids (i.e., sweat, urine, nasal secretion, and skin) were denatured with adaptations [17], [18]: the sample was placed in 345 µL RLTplus buffer mix (including, 300 µL RLT, 40 µL 1 M DTT, 5 µL Qiagen proteinase K solution) and 5 µL Carrier-RNA (1 µg/µL) in an Eppendorf tube, incubated at 37°C for up to 2 hrs for improved extraction of challenging samples.

### 4. DNase treatment

On-colum DNase I digestion using RNase-free DNase set (Qiagen) to was performed for all RNA extracts to remove DNA contamination according to manufacturer's instruction.

### 5. RNA quantification

All RNA extracts were quantified with the Quant-iT RNA assay kit and the Qubit fluorometer (Invitrogen), according to the manufacturer's protocol.

### 6. cDNA synthesis

All RNA samples were subjected to reverse transcription (RT) using the TaqMan Reverse Transcription Reagents (Applied Biosystems), random primer/oligo d (T) 16 protocol, according to the manufacturer's instructions. cDNA was obtained in a final volume of 20 µL. 1–9 µL RNA extracts were utilized in the reverse transcription reactions in order to obtain a desired quantity. For those samples with insufficient quantities, a maximum volume of extract (depending on the RT kit) was used. Negative controls (water) and positive controls (containing with the RT kit) were included all the time. Initially, to identify possible genomic DNA contamination, RT minus controls without reverse transcriptase was also performed. In sensitivity testing, serial dilutions of RNA were prepared and subjected to reverse transcription.

### 7. Body fluid markers and PCR amplification

#### 7.1 Marker selection

A candidate gene approach was used to select body fluid specific markers for study. Markers which were known to be associated with the body fluids were selected.

In view of the predominant presence erythrocytes in the blood, we choose three red blood cell relevant genes HBB, GLY and PBGD as markers for blood. GLY is a major sialoglycoprotein of the human erythrocyte membrane and carries the M or N blood group antigen [14]. HBB functions in red blood cells (erythrocytes which are around 700 times more abundant than leukocytes) as one of the globins that make up hemoglobin. PBGD has a nuclear role in addition to its cytosolic enzymatic activity required for heme synthesis [5].

Tongue scrapings were used to identify oral mucosa based on the expression HTN3 and the general mucosa marker KRT4. HTN3 and STATH were chosen as markers for saliva. For nasal secretion, the markers STATH and KRT4 were selected. HTN3 is a histidine rich protein involved in the non-immune host defence in the oral cavity while STATH is a stable, acidic salivary phosphoprotein that appears to be multi-functional [29]. KRT4 is a member of the keratin family and is mainly expressed in the tongue and in the suprabasal layers of non-cornified stratified epithelia [4].

For semen analysis, the markers PRM2 and TGM4 were selected. PRM2, as a sperm-specific mark, encodes protamine which substitutes histones in spermatozoa [6]. TGM4 is a marker specific for prostate.

For vaginal secretion, MUC4 encoding a major constituent of mucus and HBD1 encoding a vaginal antimicrobial peptide of the beta defensin family were selected [11].

For menstrual blood, the markers MMP7 and MMP11 were used. Matrix metalloproteinases are zinc dependent endopeptidases involved in the breakdown of extracellular matrix components and are expressed when tissue degradation and remodelling is required [12].

It has been reported that LOR and CST6 are the markers used for skin epithelial cells. LOR encodes loricrin which is a component of the cornified cell envelope found in terminally differentiated epidermal cells [30], [31]. CST6 a class of cysteine proteinase inhibitors found in a variety of human fluids and secretions, where they appear to provide protective functions [10], [31].

Dermcidin, a newly discovered human antibiotic peptide, was chosen as the marker for sweat investigation. Dermcidin was specifically and constitutively expressed in the sweat glands, and has been reported for positive identification of sweat in stains [25].

More importantly, we first included uromodulin (UMOD) as the marker for urine identification in a multiplex system. Uromodulin, also known as Tamm-Horsfall glycoprotein, is the most common protein excreted in the urine of healthy individuals [28], [32], [33].

GAPDH and 18S-rRNA were chosen as positive controls. These two markers have previously been used as endogenous controls [4].

#### 7.2 Primer and PCR amplification

Primers for PBGD, PRM2, MUC4, MMP7, KRT4, LOR, GAPDH, 18S-rRNA, GLY and TGM4 were adopted from the literature [4], [11], [14], [15]. Primers for HBB, HTN3, STATH, HBD1, MMP11, UMOD, DCD and CST6 were designed or redesigned in this study using Primer Premier v5.0. It was ensured that all primers designed/redesigned in this study overlap exon-exon-junctions or span at least one intron. The forward primers were 5′-labelled with 6-FAM.The primer sequences, concentrations and product sizes are indicated in Table 1. The housekeeping genes GAPDH and 18S-rRNA were used as endogenous controls.

**Table 1:** RNA markers and primer sequences used for human tissue identification in the 18 mRNA markers.

Gene	Body fluids	[primer] µM Single/Multi	Genebank accession number	Primer sequence (5′to3′)	Size(bp)	Reference
GD	Circulatory blood	0.4/0.2	NM_000190	fw: TGG ATC CCT GAG GAG GGC AGA AG rv: TCT TGT CCC CTG TGG TGG ACA TAG CAA T	177	[11]
LY	Circulatory blood	0.4/0.3	NM_002099	fw: CAG ACA AAT GAT ACG CAC AAA CG rv: CCA ATA ACA CCA GCC ATC ACC	188	[14]
HBB	Circulatory blood	0.1/0.04	NM_000518	fw: CTG AGA ACT TCA GGC TCC TGG G rv: CAG CAA GAA AGC GAG CTT AGT G	159	*
TN3	Saliva	0.2/0.15	NM_000200	fw: TCA CAT CGA GGC TAT AGA TCA AA rv: GTG TGA TGC GGT ATG ACA AAT	134	*
STATH	Nasal secretion	0.4/0.3	NM_003154	fw: ATT GGC CCT CTA GGG TAG CA rv: AGG GCC ATA CCC ATA ACC GA	213	*
PRM2	Semen	0.2/0.15	NM_002762	fw: GTG AGG AGC CTG AGC GAA CGC rv: TTA GTG CCT TCT GCA TGT TCT CTT C	294	[11]
TGM4	Semen	0.2/0.15	NM_003241	fw: AGC CTG GGC ATC TCC TCA CTA CA rv: TGG GTC CAG TTT TTA TTG GGG TGC	103	[15]
MUC4	Vaginal secretion	0.6/0.4	NM_138297	fw: GGA CCA CAT TTT ATC AGG AA rv: TAG AGA AAC AGG GCA TAG GA	235	[11]
HBD1	Vaginal secretion	0.8/0.4	NM_005218	fw: AGA TGG CCT CAG GTG GTA AC rv: GTC ACT CCC AGC TCA CTT GC	170	*
MMP7	Menstrual blood	0.05/0.025	NM_002423	fw: GAA CAG GCT CAG GAC TAT CTC rv: TAA CAT TCC AGT TAT AGG TAG GCC	126	[4]
MMP11	Menstrual blood	0.6/0.4	NM_005940	fw: ACC TTT ACT GAG GTG CAC GAG rv: CAA ATT CAT GGG CTG CCA CC	223	*
KRT4	Oral mucosa	0.05/0.015	NM_002272	fw: AAA GTC CGG ACG GAA GAG rv: TAA GAA CTG CAC CTT GTC G	81	[4]
UMOD	Urine	0.8/0.5	NM_003361	fw: GGG ACA GTG TTG ACG AGG AA rv: ATG AGC AGT GCA AAT CGG GA	341	*
DCD	Sweat	0.6/0.4	NM_053283	fw: TTT GGT GGC ATA CCC ACT CC rv: CTT TGG TGC CTG TCT GGC TA	204	*
LOR	Skin	0.8/0.4	NM_001264	fw: CTT TGG GCT CTC CTT CCT rv: AGA GGT CTT CAC GCA GTC	89	[4]
CST6	Skin	0.8/0.4	NM_001323	fw: CAC GTC GAC CTC ACC ACT TG rv: GCC CTC GGG GAC TTA TCA CA	147	*
GAPDH	House-keeping	0.2/0.2	NM_001256799	fw: GTC CAC TGG CGT CTT CAC CA rv: GTG GCA GTG ATG GCA TGG AC	261	[4]
18SrRNA	House-keeping	0.025/0.025	NR_003286	fw: CTC AAC ACG GGA AAC CTC AC rv: CGC TCC ACC AAC TAA GAA CG	110	[4]

*Original designed/redesigned for this study using Primer Premier v5.0.

In the multiplex PCR, up to 7.5 µL cDNA was amplified in the presence of 12.5 µL 2×QIAGEN Multiplex reaction mix and 5 µL 5×primer mix, resulting in a total volume of 25 µL. A GeneAmp9700 PCR System (Applied Biosystems) was used with the following cycling conditions: 95°C for 11 min, 30 cycles of 94°C 30 s, 57°C 60 s, 72°C 60 s and a final step at 60°C for 45 min.

### 8. Capillary electrophoresis (CE) and analysis

PCR products were analyzed by capillary electrophoresis on a laser-induced fluorescence ABI Prism 3130xl Genetic Analyzer (Applied Biosystems). For each reaction, 1 µL of PCR product was added to 9.5 µL of master mix (9.25 µL Hi-Di formamide and 0.25 µL GeneScan 500 LIZ Size Standard (Applied Biosystems)). A blank composed of nuclease-free water and 9947A was included as a control. Samples were processed using the default run module Fragment Analysis36_POP7_1 and dye set G5 (POP-7 Polymer, 23 second injection, 1.2 kV injection voltage, 60°C, run time 20 min, filter set G5). Raw data were analyzed using GeneMapper Software Version 3.2 (Applied Biosystems). A peak detection threshold of 100 RFUs was used.

### 9. Ladders generation

Primers without fluorescence-label were used to generate PCR templates. A total of 18 singleplex PCR were performed separately for each target body fluid, followed by plasmid standards cloning using common laboratory methods. After conformation via sequencing, all the recombinant plasmids were amplified with fluorescence-label primers. The PCR products were diluted and mixed in appropriate portions to get a balanced peak values. Bins and panels are available on demand.

### 10. Realtime PCR

cDNA was amplified using pre-designed primer assays (Table 1) and the Taqman Universal PCR Master Mix (Applied Biosystems) in a total reaction volume of 20 µL. The cDNA input amount was 1–9 µL or the same amount of H_2_O as non-template control. 18S-rRNA was used as endogenous control. The thermal cycling conditions were 95°C 5 min, followed by 40 cycles of 95°C 10 s, 57°C 45 s. For data analysis the threshold was manually set to 0.200. The amplification products were detected with a 7500 sequence detection system (Applied Biosystems). Each sample was normalized to the endogenous control, and delta Ct (dCt) was calculated by subtracting the threshold cycle (Ct) value of the 18S-rRNA endogenous control from the Ct value of the target gene. A small dCt indicates a high expression of the respective target transcript; a large dCt indicates a low or under detectable expression level.

### 11. DNA-extraction, amplification, quantification and detection

DNA was co-extracted with the AllPrep DNA/RNA Mini kit (Qiagen) and eluted in 50–100 µL EB buffer. For some Low-Copy Number (LCN) samples, elution was additionally concentrated with Microcon-30 centrifugal filter devices (Millipore) to 20 µL.

DNA extracts were measured using the Quantifiler system (Applied Biosystems), with an ABI 7500 real-time PCR machine, according to the manufacturer's instructions.

Two microliters DNA (or up to 10 µL, if the 2 µL result was weak) were amplified with the Identifilerplus multiplex Kit (Applied Biosystems) in a total reaction volume of 25 µL on a GeneAmp PCR System 9700 (Applied Biosystems) according to the manufacturer's protocol.

PCR products were detected same as 2.8.

### 12. Statistical analysis

Unless otherwise stated, in the figures, bars represent means ± standard deviations (SD) and averages were compared using a two tailed Student's t-test assuming unequal variances with a 5% significance level (*, *P*≤0.05; **, *P*≤0.01; ***, *P*≤0.001).

## Results

### 1. Positive PCR amplification results were obtained for all ten cell typing markers

**Figure 1:**
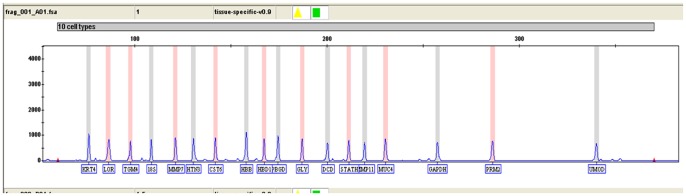
Genotyping profile of the 18 markers ladder.

According to the primer sets list (Table 1), fragments with the expected size were amplified for each of the tested body fluids or tissues and verified by sequencing (data not shown). All negative control samples, including no Reverse Transcriptase (-RT) samples, were found to be blank as expected. The same ladder shown in Fig.1 was used.

### 2. Specificity

#### 2.1 Body fluids or tissues specificity

Marker specificity of the above described 16 mRNA markers assessed in their respective body fluids or tissues collected from 4 to 16 individuals (sample collection was described in 2.2.1).

Consistent with previous studies, STATH and HTN3 were constantly expressed in saliva (Table 2) [6], [11], [13], [14]. However, STATH was less expressed compared to HTN3 in saliva in contrast to the findings of the literature [4]. Nevertheless, STATH signals were detected in nasal secretion for all tested individuals, with occasional low expression of the blood markers HBB (Table 2). The expression of HTN3 was undetectable in all examined nasal samples. KRT4, as a general mucosa marker, was detected in saliva, oral mucosa, vaginal secretion, menstrual blood, skin, female urine and nasal secretion samplings, but not in blood, semen and sweat (Table 2).

**Table 2:** Results showing specificity of the 18 mRNA markers used in various body fluids or tissues by endpoint PCR singleplexes and real-time PCR.

	Body fluid									
Body fluid Markers	Blood	Saliva	Semen	Vaginal secretion	Menstrual blood	Oral mucosa	Urine	Nasal secretion	Sweat	Skin
HBB	++	−	−	−(80%)	++	−	+	−(87.5%)	−	−
GLY	+	−	−	−	(+)	−	−	−	−	−
PBGD	(+)	−	−	−	−	−	−	−	−	−
STATH	−	+	−	−	−	(+)(75%)	−	+	−	−
HTN3	−	++	−	−	−	+	−	−	−	−
PRM2	−	−	++	−	−	−	−	−	−	−
TGM4	−	−	++	−	−	−	+(m)	−	−	−
MUC4	−	−	−	+	+	−	+(f)	−	−	−
HBD1	−	−	−	+	+	(+)	(+)(f)	+	−	(+)
MMP7	−	−	−	−(80%)	+	−	+	−	−	−
MMP11	−	−	−	−	+	−	−	−	−	−
KRT4	−	+	−	+	+	++	+(f)	+	−	+
UMOD	−	−	−	−	−	−	+	−	−	−
DCD	−	−	−	−	−	−	−	−	+	−
LOR	−	(+)	−	+	+	+	(+)	+	(+)	+
CST6	(+)	+	(+)	+	(+)	+	(+)	(+)	(+)	+
GAPDH	+	(+)	+	+	+	+	(+)	(+)	(+)	+

For all markers consisting of 4–16 individuals(as described in 2.2.1) were analyzed. Three separate experiments were analyzed. Initial total RNA was 1 ng.

1. ++ means dCt<10, peak heights>4000 RFU; + means 10≤dCt≤20, peak height 500≤RFU≤4000;

(+) means 20<dCt≤23, peak height 100≤RFU<500; - means dCt>23, peak height <100 RFU;

2. m = adult male; f = female;

3. (Percentage) indicated the percentage of individuals showed positive or negative results of the marker in the tested body fluids or tissues;

4. ‘+’ or ‘−’ means 100% positive or negative for that marker.

For vaginal secretion, in contrast to the results in previous studies that both vaginal secretion markers MUC4 and HBD1 were tested positive for saliva and oral mucosa [34], [35], MUC4 expression was only observed in vaginal secretion and menstrual blood samples (Table 2). However, HBD1 expression could be found not only in vaginal secretion and menstrual bloods but in oral mucosa, nasal secretion, skin and urine as well (Table 2).

The blood markers were also detected in the menstrual blood samples (Table 2). Among the three blood markers HBB, GLY and PBGD, GLY showed relatively low signals, while PBGD was not detected in these samples. The menstrual blood marker MMP11 showed high specificity for menstrual blood as it was not detected in any other tissue (Table 2). These findings were consistent with those described by Lindenbergh *et.al* [4]. In our results, MMP7 showed the highest level of cross-reactivity with urine samples although MMP7 seemed to be more sensitive since higher signals were obtained when compared to MMP11 (Table 2).

To assess the performance of the skin biomarkers, we analyzed two previously reported mRNA markers: LOR and CST6 [10]. Both markers showed strong signals in skin cells for nearly all tested donors (Table 2). However, these two skin markers were not observed or occasionally shown in blood and semen, but constantly highly expressed in all mucosal cells or tissues and low expressed in saliva, urine and sweat (Table 2).

For sweat identification, as illustrated in Table 2, the marker DCD was exclusively detected with generally high expression levels in sweat samples. Despite the levels of detection were moderately different between individuals, DCD produced strong signals in the sweat samples from all participants.

For urine samples, the marker UMOD showed a high level of specificity with medium-sensitivity. However, MMP7 and HBB were expressed in quite high quantities in all tested urine samples. Results variation among the individual donors was observed, namely, urine identification varies with gender and age. TGM4 was only detected in the urine of adult males whereas MUC4, HBD1 and KRT4 were only detected in female urine samples (Table 2).

The most robust positive control marker was 18S-rRNA as signals were observed in all samples. GAPDH marker was also found in all blood, semen, skin, oral mucosa, vaginal secretion and menstrual blood samples, but low expression of this marker was found in saliva, nasal secretion, sweat and urine samples (Table 2).

#### 2.2 Species specificity

In this study, we assessed the potential to detect cDNA from 8 common animal blood samples (10 ng each from pig, goat, cattle, chicken, fish, dog, cat, and mouse). The 18S-rRNA housekeeping gene was detected in all animal samples. However, no animal samples tested gave any results with the body fluid specific markers (data not shown).

### 3. Sensitivity

The sensitivity of the methods was tested with a quantitative approach (input total RNA). A dilution series of manually extracted RNA (25, 5, 1, 0.2, 0.04 and 0.008 ng) from body fluid samples except skin, urine and sweat samples was reverse transcribed and amplified. RNAs extracted from skin, urine and sweat samples were diluted as 1, 0.2, 0.04 and 0.008 ng and reverse transcribed and amplified. As we can be seen from Table 3, HBB and KRT4 appeared to be the most sensitive markers, which could detect as little as 0.008 ng RNA. HTN3, PRM2, TGM4, MMP7, LOR and CST6 were also quite sensitive and were able to detect 0.04 ng RNA. MUC4, HBD1, STATH, MMP11, GLY and DCD showed a medium sensitivity with a detection limit of 0.04–0.2 ng RNA. UMOD was less sensitive and was able to detect 0.2 ng RNA. PBGD was the least sensitive marker, which required at least 1 ng RNA.

**Table 3:** Sensitivity of the RNA profiling methods using quantitative approach (input totalRNA) by real-time PCR.

		RNA input [ng]					
Body Fluid	Marker	25	5	1	0.2	0.04	0.008
Blood	HBB	10/10	10/10	10/10	10/10	10/10	10/10
	GLY	10/10	10/10	10/10	7/10	2/10	0/10
	PBGD	10/10	10/10	7/10	3/10	0/10	0/10
Nasal secretion	STATH	8/8	8/8	8/8	7/8	4/8	1/8
Saliva		8/8	8/8	8/8	6/8	3/8	0/8
	HTN3	8/8	8/8	8/8	8/8	7/8	5/8
Semen	PRM2	4/4	4/4	4/4	4/4	4/4	3/4
	TGM4	6/6	6/6	6/6	6/6	6/6	5/6
Vaginal secretion	MUC4	5/5	5/5	5/5	4/5	2/5	0/5
	HBD1	5/5	5/5	5/5	5/5	3/5	1/5
Menstrual blood	MMP7	5/5	5/5	5/5	5/5	5/5	3/5
	MMP11	5/5	5/5	5/5	5/5	3/5	1/5
Oral mucosa	KRT4	8/8	8/8	8/8	8/8	8/8	8/8
Urine	UMOD	NT	NT	14/16	11/16	3/16	0/16
Sweat	DCD	NT	NT	6/6	5/6	2/6	1/6
Skin	LOR	NT	NT	6/6	6/6	5/6	4/6
	CST6	NT	NT	6/6	6/6	5/6	3/6

Total RNA was extracted from samples from 4–16 donors (as described in 2.2.1). A range of input total RNA was reverse transcribed and the resulting cDNA was amplified. Each experiment was carried out 3 times. dCt≤23 represent positive results. NT: not tested.

**Figure 2:**
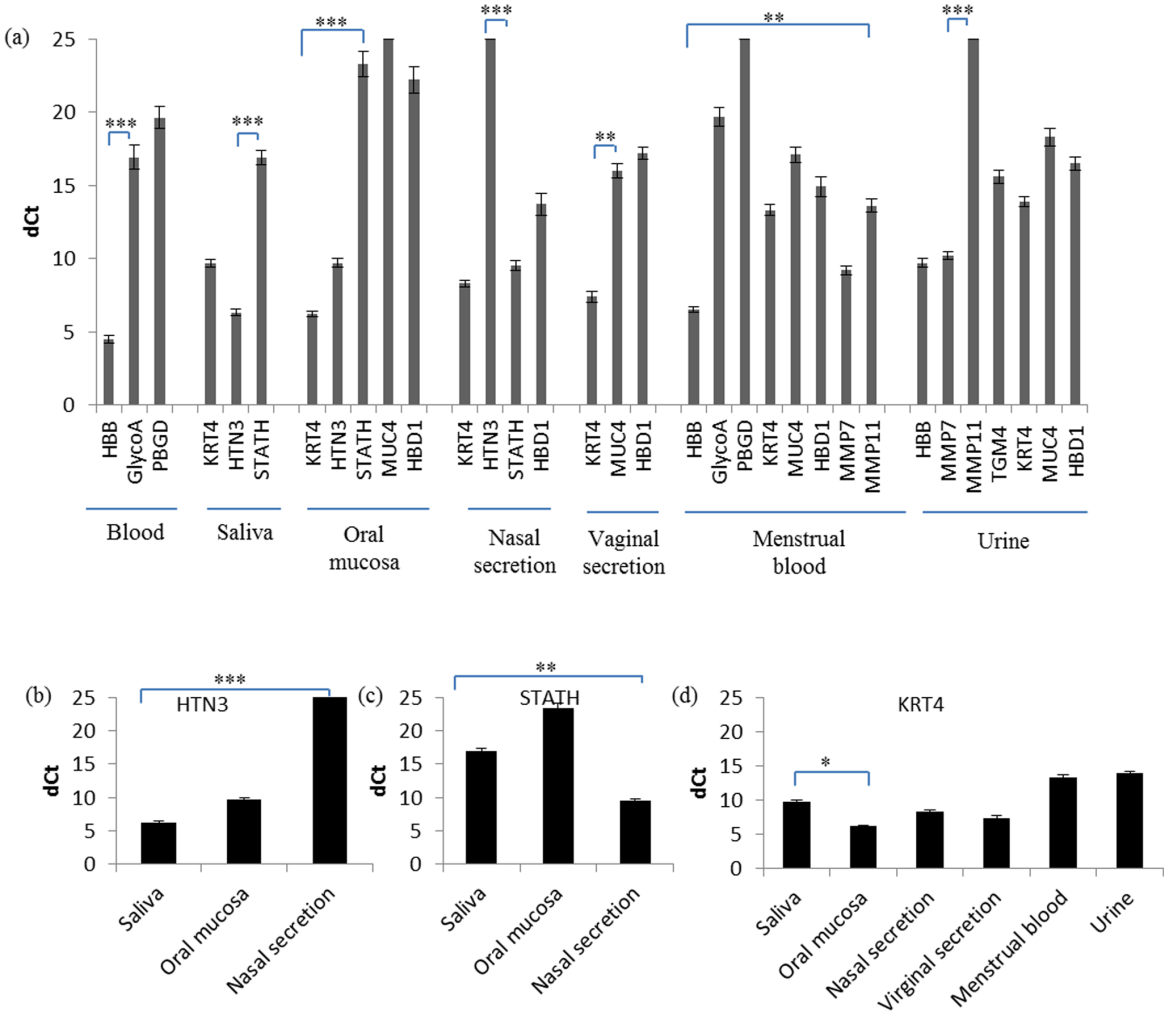
Sensitivity of cross-reactive markers in their respective body fluids or tissues. Real-Time PCR assay was used to detect sensitivity of target genes in relevant body fluids or tissues (For blood samples, n = 10; for saliva, oral mucosa and nasal secretion, n = 8; for menstrual blood samples, n = 5; for vaginal secretion samples, n = 5; for urine samples, n = 16) (a). Expression levels of target markers in each body fluid were compared with dCt values, calculated by subtracting the Ct value of 18S-rRNA (internal standard) from that of the target gene. Smaller dCt value indicates higher expression of the target gene. A dCt value of 25 indicates the cut-off value of target gene expression. Comparisons of HTN3, STATH and KRT4 mRNA levels among body fluids (b∼d). Each experiment was repeated twice. Bars represent means ± standard deviations (SD) and averages were compared using a two tailed Student's t-test assuming unequal variances with a 5% significance level (*, P≤0.05; **, P≤0.01; ***, P≤0.001).

Further, based on the specificity of each marker (described in 3.2.1 and Table 2), the detection sensitivity of the markers which showed cross-reactivity was examined in their respective body fluids or tissues using their respective primers used in this study (Fig. 2a). 18S-rRNA was used as an endogenous control. During Real Time PCR (RT-PCR), 7.5 µL cDNA was amplified for each sample. As can be seen from Fig. 2a, HBB is the highest sensitive marker in blood and menstrual blood. Likewise, KRT4 remains to be the most sensitive marker in oral mucosa and vaginal secretions. For saliva, HTN3 was found to be the highest sensitive marker.

In addition, to further examine the differences of marker expression in nasal secretions and saliva, gene expression levels for KRT4, HTN3 and STATH were also assessed in target body fluids. As shown in Fig 2b–d, KRT4 was detected in all mucous membranes with high expression levels whereas HTN3 was only highly expressed in saliva and oral mucosa. In contrast, STATH displayed highest expression in nasal secretions and medium expression in saliva, but only showed weak expression in oral mucosa, which is consist with the result of Table 2. Thus, the differences of HTN3 and STATH expression pattern could be used to distinguish between saliva and nasal secretion.

### 4. Multiplex development

An important advantage of mRNA profiling for body fluid identification is its ability to simultaneously analyze multiple markers in one multiplex PCR reaction. To develop a RNA-based characterization of different body fluids and tissues, our multiplex system (XCYR1) was designed to accommodate possible individual biological variation in gene expression levels and high sensitivities or specificities of the mRNA markers. XCYR1, including 12 markers (HBB, GLY, HTN3, STATH, PRM2, TGM4, KRT4, MUC4, MMP7, MMP11, DCD and UMOD) with two house-keeping genes (18S-rRNA and GAPDH) was developed based on the sensitivity and specificity with the optimized primer concentrations (Table 1). No extraneous peaks were observed at marker positions in -RT samples or PCR negative controls. Genotyping profile of XCYR1's ladder is shown in Fig. S1.

**Figure 3:**
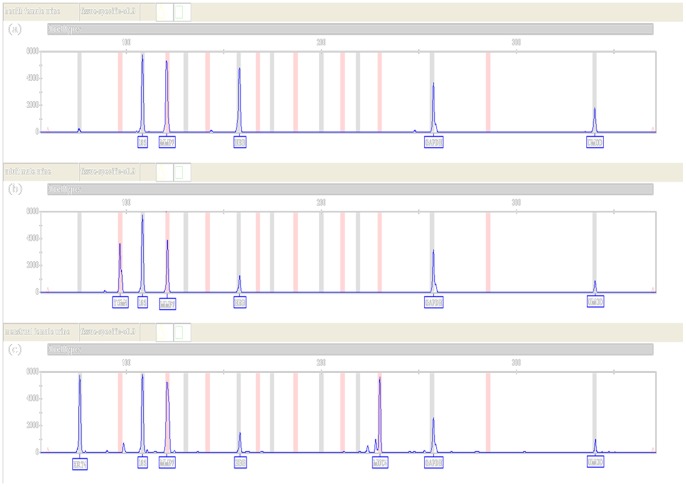
Detection of urine samples with urine specific markers using XCYR1 by CE; the same ladder as Fig.1 was used. (a) female urine samples collected during non-menstrual period; (b) male urine samples; (c) female urine samples collected during menstrual period.

As no such report is available so far to investigate the detection of urine samples with an mRNA multiplex assay, we further studied the expression of relevant urine specific markers in adult male urine samples, healthy female urine samples and female urine samples during menstrual period using XCYR1. The results of Fig. 3a–c showed that MMP7, HBB and UMOD were detected in all urine samples, which is consistent with the results in Table 2 described in the previous section. However, TGM4 was only detected in the urine of adult males (Fig. 3b), of which MUC4 and KRT4 were shown to be present at high quantity in female urine samples collected during menstrual period (Fig. 3c).

### 5. Mixtures

#### 5.1 Detection of body fluid mixtures by multiplex XCYR1

**Figure 4:**
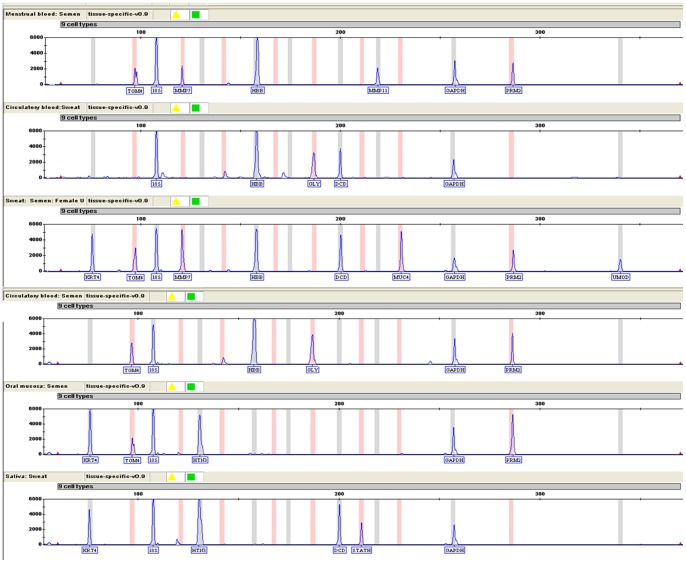
Detection of two or more-body fluid mixtures using XCYR1 by CE. (a) Menstrual blood: Semen; (b) Blood: Sweat; (c) Sweat: Semen: Female urine; (d) Blood: Semen; (e) Oral mucosa: Semen; (f) Saliva: Sweat.

Body fluids mixtures from intra- and inter- individuals were deposited on swabs (as described in Section 2.2.2). The electropherograms of body fluid mixture samples detected with XCYR1 were shown in Fig. 4a–f. All expected markers were amplified, only the blood-specific marker GLY was not detectable in the menstrual blood and semen mixture sample (Fig. 4a).

#### 5.2 Prediction of mixture ratios of blood-sweat and blood-semen mixtures

To assess whether the mixture ratios of various two-component body fluid mixture could be predicted, we performed pilot study on blood-sweat and blood-semen mixtures. Firstly, XCYR1 multiplex was used to analyze the nature of the two body fluid mixture as did in 3.5.1. Then we performed mRNA profile analysis of respective tissue fluid markers in the mixture.

**Figure 5:**
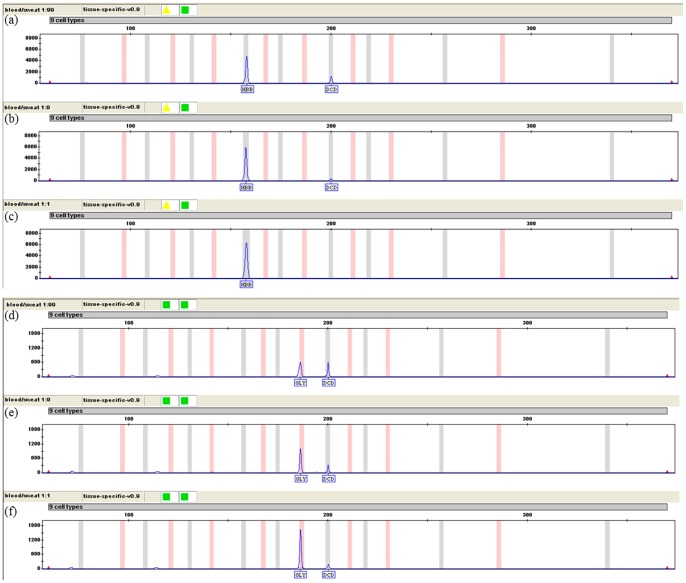
Representative CE of the mRNA multiplex of mixed body fluids (blood: sweat). (a) Blood: sweat 1∶99 ratio mRNA profile (HBB: DCD). (b) Blood: sweat 1∶9 ratio mRNA profile (HBB: DCD). (c) Blood: sweat 1∶1 ratio mRNA profile (HBB: DCD). (d) Blood: sweat 1∶99 ratio mRNA profile (GLY: DCD). (e) Blood: sweat 1∶9 ratio mRNA profile (GLY: DCD). (f) Blood: sweat 1∶1 ratio mRNA profile (GLY: DCD). A total volume of 30 µL was used for detection. Each experiment was repeated twice.

Fig. 5a–f showed the results of mixtures of blood and sweat in 1∶99, 1∶9 and 1∶1 ratios. With a 1∶99 ratio blood and sweat mixture, the peak height ratio of HBB/DCD and GLY/DCD were about 5∶1 (Fig. 5a) and 1∶1 (Fig. 5d), respectively. With a 1∶9 ratio blood and sweat mixture, the HBB/DCD and GLY/DCD peak height ratio were approximately 15∶1 and 3∶1, respectively (Fig. 5b and e). With a 1∶1 ratio blood and sweat mixture, the GLY/DCD peak height ratio was 9∶1 (Fig. 5f) whereas Fig. 5c (HBB/DCD) only indicated the marker of blood (HBB). Consequently, the ratio of GLY/DCD is closer to the ratio of body fluids deposited than that of HBB/DCD, indicating GLY and DCD are the most suitable markers to detect blood and sweat mixtures ratios.

**Figure 6:**
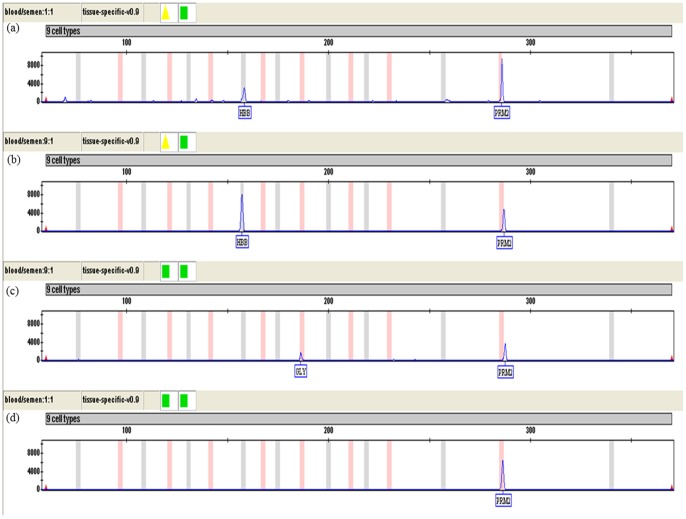
Representative CE of the mRNA marker of mixed body fluids (semen: blood). (a) Semen: blood 1∶1 ratio mRNA profile (PRM2: HBB). (b) Semen: blood 1∶9 ratio mRNA profile (PRM2: HBB). (c) Semen: blood 1∶9 ratio mRNA profile (PRM2: GLY). (d) Semen: blood 1∶1 ratio mRNA profile (PRM2: GLY). A total volume of 30 µL was used for detection. Each experiment was repeated twice.

The test results of semen and blood mixture (1∶1 and 1∶9 ratio) were shown in Fig. 6a–d. Peak height of PRM2/HBB was approximately 3∶1 and 1∶2 when semen and blood were mixed at 1∶1 and 1∶9 ratios (Fig. 6a and 6b). On the other hand, when using PRM2 and GLY as tissue specific markers, PRM2/GLY peak height ratio was about 2∶1 for 1∶9 semen and blood mixture whereas only PRM2 was detected for 1∶1 semen and blood mixture (Fig. 6c and 6d). Thus, PRM2/HBB is a more suitable parameter than PRM2/GLY to indicate the ratio of blood and semen mixture deposited.

### 6. Forensic casework/mock samples

RNA and DNA were extracted from 30 forensic casework and 6 mock samples. RNA profiling was done with XCYR1 assay. As illustrated in Table 4, all samples were also analyzed by STR-profiling before mRNA profiling. Full DNA profiles of each donor consistent with the reference profiles of the donor were obtained for all except for some samples (these exceptions gave partial or no profiles).

**Table 4:** Identification of 30 forensic casework and 6 mock samples with XCYR1.

Sample	XCYR1												STR
	HBB	GLY	HTN3	KRT4	STATH	MUC4	MMP7	MMP11	PRM2	TGM4	DCD	UMOD	
**Forensic casework (Blood)**													
wood	+	(+)											14
wall	+												n.d.
tissue	+	+											16
knife edge	+	+											16
cloth	+	+											16
sanitary towel	+	+					+	+	+	+			16(M)
**Forensic casework (Saliva)**													
stamp													n.d.
tissue			+	+	+								16
bottle			+	+									15
cigarette			+	+									16
chewing gum	+	(+)	+	+	+								16
chopstick			+	+	(+)								15
**Forensic casework (Semen)**													
bed sheet				+		+			+	+			16(M)
tissue									+	+			16
swab from vagina				+		+							16
underpants				+		+							16
condom inside										+		(+)	10
**Forensic casework (Vaginal secretion)**													
bed sheet				+		+							16
swab from vagina	+			+		+	+	(+)	+	+			16(M)
condom outside				+		+							16
underpants				+		+			+	+			16
**Forensic casework (Sweat)**													
undershirt armpit											+		14
undershirt washed													n.d.
socks toe											+		12
socks center													7
knife handle	+	+									(+)		16(M)
cap inside margin											+		15
rubber glove palm											+		16
tissue			+	+	(+)						+		12
mouse													6
**Mock sample**													
1. Menstrual blood and semen on the towel	+	(+)		+			+	+	+	+			16(M)
2. Nasal secretion and blood on sanitary cotton	+	+		+	+								16
3. Saliva and semen on the issue			+	+	(+)				+	+			16
4. Swab from blood on the sweaty-forehead	+	+									(+)		16
5. Swab from saliva on the sweaty-neck			+	+	(+)						+		16
6. Sweat and urine on the swab											+	(+)	11(M)

1. The number of analyzed loci in DNA profiles from coextracted DNA (from the same piece of stain) are shown. 16 loci represent a full Identifilerplus profile. (n.d. =  not detected. M =  mixture STR result).

2. +: peak heights are above 150 RFU; (+): peak heights are 100–150 RFU; blank: peak heights are below 100 RFU.

Blood on wood, which contained low amounts of DNA, produced a partial DNA profile but a full RNA profile. This may be due to the presence of inhibitors or sufficient recovery. For a particular sample, a 2-year-old blood stain on wall, only one of the blood mRNA markers was detected (HBB) but no DNA profile could be recovered. This could be due to the very low amount of nucleated cells compared to the large amount of reticulocyte present in the sample.

For the stain on the back of stamp, which probably contained saliva or sweat, no RNA and DNA profile was amplified, ruling out the possibility of the presence of any biological stain in this sample. Partial male STR profiles and TGM4 peak were obtained from the semen sample, but the semen targeted RNA marker (i.e., PRM2) was not amplified. This probably is because the sample may also contain exfoliated cells. For vaginal secretion sample (vaginal swab), both marker for female vaginal secretion (MUC4) and markers for semen (PRM2 and TGM4) were detected (Table.4). It implies that this sample might have sexual contact prior to sample collection. DNA STR profile also confirmed that it was a mixed male and female STR profiling.

For mock sweat samples, when blood was swabbed from the sweaty-forehead after prick, blood markers were detected with high signals but sweat marker was only detected when using a high cDNA input. For saliva samples collected from the sweaty-neck swab, markers for both saliva and sweat were detected but saliva signals were predominant strong compared to the sweat marker signal (data not shown). This is probably due to the number of different cell types per unit area in mixture samples.

Finally, interpretation guideline of the mRNA profiling results obtained from XCYR1 assay was summarized in Table 5. Both the presence and the absence of markers' peaks should be considered in the interpretation.

**Table 5:** Overview of XCYR1 results per cell type to guide RNA profile interpretation.

Body fluids/cell types	XCYR1											
	HBB	GLY	HTN3	STATH	KRT4	MUC4	MMP7	MMP11	TGM4	PRM2	DCD	UMOD
Blood	+	+										
Saliva			+	+	+							
Oral mucosa			+	(+)	+							
Nasal secretion				+	+							
Vaginal secretion					+	+						
Menstrual blood	+	(+)			*	*	+	+				
Semen									+	Δ		
Sweat											+	
Urine	+				*	*	+		#			+

(+) very low expression.

*possible expression.

# adult male only.

Δ fertile men only.

## Discussion

This study sought to evaluate the specificity and sensitivity of 18 mRNA markers by capillary electrophoresis and real-time PCR for the identification of ten human body fluids and tissues of forensic interests. We for the first time successfully incorporated the identification of urine, sweat and nasal secretion into a single multiplex system. Our results demonstrated DCD was a highly specific and sensitive marker for sweat identification. UMOD specifically expressed, but at medium level, in urine samples whereas HBB and MMP7 had unexpected high expression in human urine. TGM4 had a high expression level in adult male urines. However, the expression of KRT4 and MUC4 varied with individuals in female urines. Our results also showed that although STATH could be found in nasal secretion, saliva, and occasionally in oral mucosa, another marker HTN3 which was specific for saliva and oral mucosa identification was not expressed in nasal secretion. Finally, we have successfully developed a multiplex system called XCYR1which incorporates 14 mRNA markers targeting from nine human body fluids and tissues in one PCR reaction. XCYR1 has shown excellent sensitivity and specificity in forensic casework identification.

HTN3 and STATH were two markers commonly used for saliva identification [4], [6], [14]. Using redesigned primers for HTN3 and STATH in this study, both large histatin isoform (HTN1) and small STATH isoform described by J. Juusola *et al.* [11] were not observed here. In some occasions, blood specific markers were detected in saliva samples (Table 4). This was probably due to small amount of blood in the saliva samples. As shown in the Fig 2a, detection of HTN3 was much more sensitive than STATH in saliva. These findings were slightly different from previous studies, in which both HTN3 and STATH had high levels of specificity and sensitivity in saliva [6], [11]. In this study, the expression of STATH was relatively high in nasal secretion whereas its expression remained quite low expression in saliva and oral mucosa (Fig 2c). In contrast, HTN3 was highly expressed in saliva and oral mucosa but no expression was detected in nasal secretion (Fig 2b). Tongue scrapings were used to assess the expression of the mucosa markers in tongue cells which can be found on licked items. The tongue swab samples should have a higher proportion of oral mucosal cells than saliva. Moderate expressions of STATH and HTN3 were detected, probably because the samples contained saliva. Consistent with the findings of Sakurada *et al*. [36], [37], in our results, STATH not only present in saliva but is highly expressed in nasal secretion (Fig 2c). However, HTN3 was only detected in saliva, and STATH was not expressed in vaginal secretion in our study (Table 2). Therefore, the difference of the STATH and HTN3 expression levels could be used to differentiate nasal secretion from saliva or oral mucosa by real-time PCR and multiplex assays.

Consistent with Sakurada *et al.* [25], DCD was detected only in the sweat stains but not in any other body-fluid stains (Table 2), as DCD was specifically and constantly expressed in the sweat glands, but not in epidermal keratinocytes [23], [24]. In our study, DCD was incorporated, for the first time, into a multiplex assay and it was proved to be a highly sensitive and specific marker for sweat identification.

UMOD is expressed in renal tubules and secreted in urine [32], [33], [38]. Akutsu *et.al* firstly detected the expression of THP in urine samples by ELISA and real time PCR (RT-PCR) [28]. They showed that THP was detectable only at the protein level but difficult to detect by RT-PCR at the mRNA level. In our study, we investigated the feasibility to use UMOD for body fluids identification. Slightly different from previous studies [28], [39], UMOD expression was exclusively detected in urine samples but not in any other body fluids (Table 2). Thus, UMOD was an appropriate marker for urine identification. In addition, TGM4, specific to prostate, was detected in adult male urine samples (Fig. 4b). A possible reason may be that prostate-specific antigen (PSA, also known as p30) exists not only in semen and seminal fluid, but in adult male urine [1], [2]. On the other hand, KRT4, MUC4 and HBD1 expression were only detected in female urine, of which MUC4 was shown to be highly expressed in urine samples collected during menstrual period (Fig. 4c). However, MMP7 and HBB had a strong, constant expression in the urine of all donors. The presence of trace hemoglobin and apoptotic cells in human urine samples may explain this result. Detecting trace hemoglobin present in urine samples by RT-PCR, HBB could be more easily observed with higher sensitivity compared with other markers like GLY. Matrix metalloproteinases (MMPs) are the key effector of cell differentiation, cyclic growth and cell death of the endometrium and are regulated by ovarian steroids and cytokines [40], [41]. Previous studies have shown that MMPs play an intriguing role in apoptosis, showing both apoptotic and anti-apoptotic action [41], [42]. MMP7 can release Fas-L which binds to death receptor Fas and triggers apoptosis of adjacent cells [43]. This may explain the presence of MMP7 in the urine samples in our study. However, we are still not very clear why MMP11 was not detected in the urine samples as MMP11 was also shown to be involved in apoptosis during tissue remodeling and development [44].

MUC4 and HBD1 were previously reported to be able to identify vaginal secretion samples [4]. However, it was also reported in other studies that vaginal secretion marker MUC4 may be present in saliva stains and oral epithelial cells [6], [11], [34], [45] Although the expression level differed among donors, MUC4 was clearly and specifically expressed only in all test donors' vaginal secretion. Weak expression of the menstrual secretion marker, MMP7, and the circulatory blood marker, HBB, were also observed in some donors (Table 2&4). Our results were consistent with those of Lindenbergh *et al.* [4]. Different from the exclusive expression of MUC4 in vaginal samples, low level of expression of HBD1 was observed in other mucous samples (Table 2). Although the primers were redesigned, the expression of HBD1 was still low, perhaps due to poor multiplexing capability. Collectively, these results suggest that HBD1 may not be an ideal marker to multiplex into the assay. Therefore HBD1 was excluded from the assay development.

Previous studies have shown both MMP7 and MMP11 are excellent markers for the determination of menstrual blood [4], [6], [12]. Our study showed that MMP7 was more sensitive than MMP11 whereas MMP11 was more specific than MMP7 for menstrual blood. On the other hand, as menstrual blood is a complex mixture with multiple tissue types, blood and vaginal markers (Table 2) could have positive peaks in menstrual blood [9]. In menstrual blood samplings, GLY expression was relatively low, whereas PBGD was undetectable, probably due to mRNA degradation in blood cells caused by the microbial vaginal flora or acidic environment [4].

Although the identification of human skin cells is important in many forensic cases, the two commonly used skin specific markers CST6 and LOR showed high cross-reactivity in other body fluids in this study. Therefore, we excluded the markers for skin tissue identification from our multiplex system. On the other hand, the 14 markers incorporated in our multiplex system showed excellent capacity in identifying specific body fluids or tissues origin. Skin cells, which may be unavoidable during sampling of some body fluids or tissues such as sweat or mucosa, did not show any interference in our multiplex system.

The mRNA-profiling multiplex technique shows promise as a method that can detect body fluids in mixed ratios [14], [46]. Since different body fluids contain different numbers of nucleated cells per unit area varying both inter- and intra- individuals, it is always useful and meaningful to choose suitable markers based on the types of body fluids mixture (differences in numbers of cells per unit volume or area).To analyze an unknown body fluid mixture, it is necessary and meaningful to first use an mRNA-profiling multiplex to find out the nature and component of the mixture. Then appropriate primers could be chosen to detect the ratio of different body fluids in the mixture. For example, for blood and sweat mixture, the blood identification marker with relatively low mRNA expression level should be chosen. In contrast, when detecting the mixture of semen with a small amount of blood, high mRNA expression marker should be chosen for blood identification.

Several factors including time post sample collection, age and individual variation may potentially influence the detection efficacy of XCYR1. Although mRNA is highly unstable and rapidly degraded by ubiquitous RNases, several studies have clearly shown mRNA in stains is highly stable not only under controlled conditions but when exposed to a range of environmental conditions [3]–[6]. In this study, we have successfully detected HBB expression by XCYR1 in a 2-year-old blood stain on wall, which is in agreement with previous studies (Result section 3.7). In addition, no significant age-dependent difference and individual variation was found in adult volunteers except for the identification of urine. The result of urine identification varies with gender and age. Our results showed that TGM4 was only detected in the urine of adult males whereas MUC4, HBD1 and KRT4 were only detected in female urine samples

In summary, the results of this study support the use of mRNA profiling for the positive identification of the vast majority of forensically relevant biological fluids or tissues. Targeting samples from nine different cellular origins, XCYR1 shows excellent sensitivity and specificity in forensic casework. However, further work is required to seek new candidates and include more markers for the identification of vaginal secretion, skin, sweat, tear and urine stains. For example, CYP2B7P1 for vaginal secretion [19], [47] and LCE1C for skin [17], [19], [33] should be considered in future multiplex development. Other more sensitive markers would contribute to the reliable identification of LCN samples in forensic casework. Challenges in the future work will be to study the extremely environmental-exposed biological stains identification to outdoor crime scenes by mRNA profiling.

## Supporting Information

Figure S1 **Genotyping profile of the XCYR1's ladder.**
(TIF)
