# Correction: Development of Highly Sensitive and Specific mRNA Multiplex System (XCYR1) for Forensic Human Body Fluids and Tissues Identification

**DOI:** 10.1371/journal.pone.0105448

**Published:** 2014-08-06

**Authors:** The PLOS ONE Staff

Figure 3 is illegible; the publisher apologizes for the error. Please see the corrected Figure 3 here.

**Figure 3:**
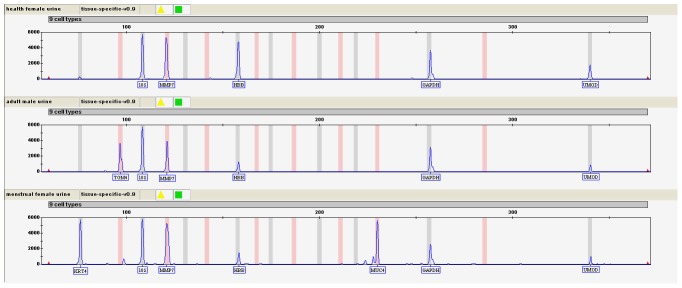
Detection of urine samples with urine specific markers using XCYR1 by CE; the same ladder as Fig. 1 was used. (a) female urine samples collected during non-menstrual period; (b) male urine samples; (c) female urine samples collected during menstrual period.
