# Supplementary figures and images for: Development of Highly Sensitive and Specific mRNA Multiplex System (XCYR1) for Forensic Human Body Fluids and Tissues Identification
Source: PLoS One. 2014 Jul 3;9(7):e100123. doi: 10.1371/journal.pone.0100123 (PMC4089028; doi:10.1371/journal.pone.0100123)

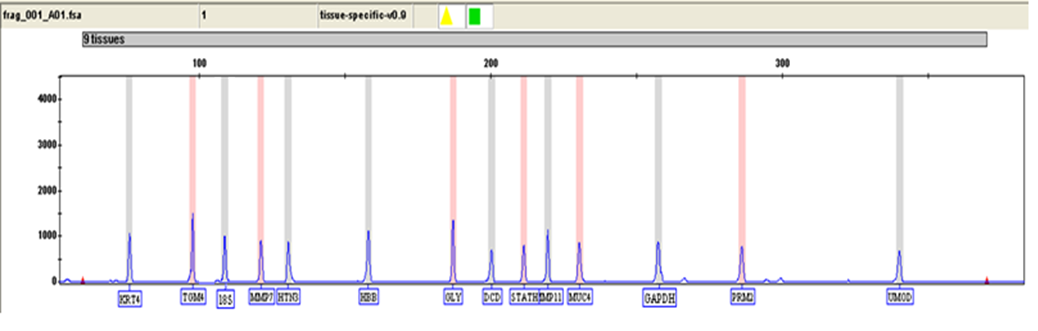

Supplement: Figure S1 — Genotyping profile of the XCYR1's ladder. (TIF) [file pone.0100123.s001.tif]
